# The impact of coworker guanxi on workers’ safety behaviors in the construction industry: the effects of team identification, team knowledge sharing, and team safety climate

**DOI:** 10.3389/fpubh.2025.1659728

**Published:** 2025-09-10

**Authors:** Lixia Wang, Xun Luo, Hujun Li

**Affiliations:** ^1^School of Business Administration, Henan Polytechnic University, Jiaozuo, China; ^2^School of Civil Engineering, Henan Polytechnic University, Jiaozuo, China

**Keywords:** coworker guanxi, workers’ safety behaviors, team identification, team knowledgesharing, team safety climate, construction workers

## Abstract

This study explores how coworker guanxi (CG) influences workers’ safety behaviors (WSBs) in China’s construction industry, focusing on the mediating roles of team identification (TI) and team knowledge sharing (TKS), and the moderating effect of team safety climate (TSC). Based on social exchange theory and previous literature, the research proposes a dual-mediation model to capture the complex interplay between these factors. A survey was conducted with 347 frontline construction workers across three major Chinese cities to test the hypotheses. Latent variable models revealed that CG has a significant positive impact on WSB, both directly (*β* = 0.155, *p* < 0.001) and indirectly through TI (indirect effect = 0.216) and TKS (indirect effect = 0.230). The results also showed that TSC moderates the relationship between CG and both TI and TKS, amplifying the positive effects on safety behaviors in construction teams. These findings offer important theoretical contributions by integrating CG into safety behavior research and extending social exchange theory in the context of Chinese construction environments. Additionally, the study provides practical insights for construction managers, suggesting that fostering strong coworker relationships and a positive safety climate can significantly improve safety behaviors, ultimately reducing workplace accidents and enhancing overall safety performance.

## 1 Introduction

In recent years, governments at each administrative level in China have vigorously promoted infrastructure development, strengthening the importance of the construction industry as a key driver of the national economy. However, the industry still grapples with ongoing safety issues (1) due to its inherently dynamic work environments, complex multi-tier subcontracting systems, poor on-site safety management practices, and heavy reliance on a transient labor force (2). As a result, construction-related accidents remain frequent, often leading to substantial economic, societal, and personal losses (3). A growing body of research has identified unsafe behaviors among construction workers as a primary contributing factor to these incidents (4). Accordingly, a central and ongoing challenge for the construction industry is determining how to effectively foster workers’ safety behaviors (WSBs) and thereby reduce the incidence of occupational accidents.

Existing research indicates that WSBs emerge from complex, multi-level interactions involving social, organizational (or team), and individual factors in construction settings (5). However, much of the current literature has adopted a formal contract-based approach to safety behavior governance, which often overlooks the guanxi aspects that are particularly salient in Chinese construction settings (6, 7). Rooted in Chinese traditional culture, guanxi is a specific, personal bond that enables the exchange of favors between the parties involved (8, 9). Specifically, whether workers adhere to safe practices is not solely a function of formal rules and oversight, but is also significantly shaped by informal interpersonal guanxi with other participants in safety practice, especially coworker guanxi (CG). CG can be referred to the degree of trust interaction and emotional closeness between a worker and his (her) workmates (10, 11). Previous studies on CG have demonstrated that CG is a salient predictor of individual work-related psychology status [e.g., employee motivation and stay intent (12)] and work-related outputs [e.g., career success (13), low-carbon behaviors (14), charge-taking (15), knowledge sharing (16), job performance (11, 17)]. For Chinese construction workers, the influence of CG is more significant. On one hand, CGs between construction workers are mostly innate ties (e.g., kinship, marital ties, and geographical ties), these ties maintain the formation of the construction work team (18, 19). On the other hand, guanxi interactions based on CG are important for skill-learning and useful information-sharing because of the loose and temporary contractual relationships with other participants (owners and contractors) in the Chinese construction industry. Accordingly, the closeness and frequency of interaction between their workmates can meaningfully shape behavioral tendencies, including the inclination to engage in safe actions (20, 21). Yet, only a few scholars have started to pay attention to the impact of CG on WSBs. Chen et al. (22) argued that there exists a positive relationship between CG and WSBs, and the team identification (TI) can be a mediator. However, this study still has limitations: (a) the study regards the CG as a single-dimensional concept, while similar to other types of guanxi, the CG should be multi-dimensionally conceptualized when testing its predictive power; (b) the influence process of the research is somewhat single, but the influence of CG on WSBs is complex, some other mediators and moderators should be incorporated to explain this mechanism. Therefore, it is necessary to elaborate on the conceptualization of CG and further explore the complex influencing mechanism of CG on WSBs.

Except for the mediating role of TI in the influencing mechanism, as argued by Chen et al. (22), two other factors that appear particularly relevant in this context are team knowledge sharing (TKS) and team safety climate (TSC). In work environments where team members often share familial or pseudo-familial ties (19), construction workers frequently rely on peer communication to acquire safety-related knowledge (23, 24). Strong interpersonal ties can facilitate the efficient dissemination and exchange of both tacit and explicit safety knowledge (25, 26), thereby equipping workers with the necessary information to make informed, safe decisions on site. TSC refers to the workers’ shared perception to the extent how management staff prioritize onsite safety in the work team (27, 28). We argued that closer CG might promote the sharing of safety knowledge among workers and is more conducive to the generation of team identification in a high level of TSC due to the attention of the organizational or group management personnel (29). Thus, TSC might influence the process of CG affecting WSBs. Accordingly, this study integrates TI, TKS, and TSC into its analytical framework to investigate how CG shape WSBs in the construction industry.

Drawing upon established theoretical frameworks and existing empirical literature, this study established a conceptual model to examine the relationships among CG, TI, TKS, TSC and WSBs in construction. The model is empirically tested using data collected from frontline construction workers, with measurement scales adapted from validated scales. A questionnaire survey serves as the primary data collection tool, and the latent variable model (LVM) is employed to assess the proposed conceptual model and test the hypothesized mediating effects of TI and TKS, and the moderating effects of TSC.

The objective of this research is to elucidate the mechanisms through which CG among construction workers influences their WSBs. By investigating both the direct, indirect and moderating pathways of influence, this study aims to offer a deeper insight into the social factors that influence safe behavior on construction sites. The findings are expected to contribute to the theoretical advancement of safety behavior research, particularly within the context of relational governance in construction. Additionally, the study offers practical implications for project managers and safety practitioners, enabling the design of more effective, socially informed safety strategies to reduce accidents and improve industry-wide safety performance.

## 2 Literature review and hypotheses

### 2.1 Coworker guanxi and workers’ safety behaviors

CG can be simply defined as informal interpersonal relations between an individual and his (her) workmates (22), and often denotes the degree of trust interaction and emotional closeness within dyads. In China, this type of interpersonal relation is based on particularism (30) and functions as a mechanism for the distribution and acquisition of resources (31, 32). Given that Chinese construction workers are often organized through (pseudo) kinship ties (18, 19, 33), their guanxi with workmates tends to be closer when compared to other organization or team contexts (34). Some prior studies conceptualized CG as a unidimensional variable (13, 22). More scholars believe that it is multi-faceted: affective relation and instrumental relation (10); affective attachment, personal-life inclusion, and deference (35); affection, reciprocity, and trust (36); and affective relation, instrumental relation, obligation and face (11). It is worth noting Chen et al.’s dimension structure after reviewing the measurement scales for the aforementioned dimensions. Although designed to describe guanxi between a worker and his (her) foreman, affective attachment and personal-life inclusion in this dimension structure truly depict the guanxi interactions between workers and their workmates in the Chinese construction context. Thus, this research selected affective attachment and personal-life inclusion to interpret CG.

WSB refers to the observable actions of individuals that align with organizational safety rules, procedures, and expectations, aimed at preventing accidents and promoting a safe work environment (37, 38). It is typically conceptualized as having two components: safety compliance and safety participation. Safety compliance denotes core behaviors necessary for maintaining workplace safety, such as wearing PPE and following operational procedures, while safety participation involves proactive, voluntary actions that contribute to the broader safety culture, such as reporting hazards or assisting co-workers in risky situations (39, 40).

Existing literature in business management emphasizes that coworker guanxi (CG) plays a crucial role in shaping individual behaviors, influencing decision-making processes and outcomes (41). In the context of construction workers, CG, as a key interpersonal relationship, directly affects their safety behaviors. This is consistent with the Theory of Planned Behavior (42), which asserts that behavioral intention is a critical precursor to individual actions. CG significantly influences workers’ safety intentions, as workers with strong emotional bonds and trust are more likely to adopt safe practices. Workers are motivated not only by formal safety regulations but also by their desire to reciprocate the trust and support they receive from their coworkers, reflecting the core principles of Social Exchange Theory (SET) (43). Moreover, CG fosters social norms, emotional maintenance, and reciprocity, which significantly influence safety behaviors on construction sites. Studies indicate that CG enhances workers’ willingness to share tacit knowledge, which is crucial for improving safety practices. When workers share safety-related knowledge with their peers, they become more aware of risks and better equipped to prevent accidents (44). Furthermore, the relational dynamics within CG strengthen mutual responsibility for safety, encouraging workers to take proactive safety measures, not just for themselves but for the team as a whole (45). Based on the aforementioned argument, the following hypothesis can be drawn.

*H1*: Coworker guanxi is associated with workers’ safety behavior.

### 2.2 Mediating effect of team identification

TI is defined as the extent to which a person identifies themselves as belonging to a particular team (e.g., work team), and it reflects a cognitive, emotional, and evaluative bond between the individual and the team (46). The conceptualization of this construct can be dated back to social identity theory and self-categorization theory, which pointed out people categorize them into different teams based on the homogeneity between their self-concept and the teams’ values, goals, and norms, and thus, they develop an identification with the team (47). According to the existing literature, TI can be interpreted as a unidimensional concept or divided into three mutually-interrelated components, including cognitive identification, affective identification, and evaluative identification (48, 49). In construction work settings, where teams often operate in high-risk, interdependent environments, strong TI can enhance cohesion, promote mutual support, and motivate individuals to align their behaviors with collective goals, including safety-related expectations.

From the perspective of Social Exchange Theory (SET) (43), CG functions as a relational exchange characterized by mutual trust, emotional support, and reciprocal obligations. When individuals perceive consistent interpersonal investment from coworkers, they are inclined to respond with affective commitment, a core mechanism through which exchange relationships translate into psychological attachment to the group (50). This process is not merely transactional; rather, it generates a sense of relational obligation that motivates individuals to align their self-concept with the team (22). As CG strengthens, workers experience greater emotional safety and reduced social uncertainty, conditions that foster deeper engagement and willingness to reciprocate beyond formal job requirements (51). Over time, repeated positive exchanges accumulate into a sense of belonging, transforming interpersonal trust into collective identification. Besides, from the perspective of Social Identity Theory (52), CG strengthens TI by enhancing the psychological salience of team membership. CG fosters a shared sense of belonging through repeated in-group interactions, enabling individuals to internalize the team as part of their self-concept. As relational ties become symbolically meaningful, they reinforce in-group boundaries and reduce perceived interpersonal differentiation (53). This self-categorization process is amplified in collectivistic contexts, where close interpersonal bonds serve as identity signals that distinguish “us” from “others” (54). Even in non-Chinese settings, informal ties function similarly by transforming team membership from a structural role into a socially defined identity (55).

From the perspective of Social Identity Theory (52), TI shapes workplace safety behaviors (WSBs) by aligning individual actions with in-group norms. When workers strongly identify with their team, they internalize team-based values and standards as part of their self-concept, leading to spontaneous compliance with safety practices that are perceived as central to the group’s identity (56). This self-categorization process reduces the need for external monitoring, as individuals regulate their behavior to remain consistent with “what we do” rather than “what I am told to do” (57, 58). In high-risk environments such as construction, safety norms become symbolically linked to team membership, making adherence a marker of belonging (59). Besides, TI enhances normative regulation by increasing the salience of collective outcomes—workers who see themselves as part of the team are more likely to avoid unsafe acts that could harm fellow members or damage team reputation (33, 60). Moreover, identified team members are more responsive to peer influence, as feedback from coworkers is interpreted not as criticism but as in-group correction, thereby promoting timely behavioral adjustment (61). Recent studies further confirm that TI strengthens shared mental models of safety, enabling teams to anticipate risks and coordinate preventive actions without explicit instruction (62). Additionally, in collectivistic work contexts, TI amplifies the internalization of safety as a moral duty toward the group, where violating safety rules is seen as a betrayal of trust (63). According to previous arguments, the following hypotheses can be proposed.

*H2*: Coworker guanxi is associated with team identification.

*H3*: Team identification is associated with workers’ safety behavior.

*H4*: Team identification mediates the relationship between coworker guanxi and workers’ safety behavior.

### 2.3 Mediating effect of team knowledge sharing

TKS refers to the process through which team members share and disseminate valuable information, expertise, and skills within the team (64, 65). This includes both explicit knowledge (e.g., documented procedures or technical data) and tacit knowledge (e.g., personal experiences or insights from practice) (66, 67). Some previous literature has identified two common components for TKS: explicit and tacit knowledge sharing (68). Some other scholars view it as a three-dimensional concept, consisting of cognitive, motivational, and behavioral components (69, 70). On construction sites, TKS is crucial for enhancing safety by ensuring efficient communication and implementation of safety knowledge. Effective knowledge sharing improves decision-making, reduces risks, and boosts team performance, and thus, provides workers with the necessary safety information and expertise to prevent accidents and improve overall performance.

From the perspective of Social Exchange Theory (SET), CG, characterized by emotional bonds and mutual trust, facilitates team knowledge sharing (TKS) by reducing transaction costs and social risks (43). SET posits that individuals engage in exchanges based on reciprocity and fairness (71). In construction teams, CG fosters generalized reciprocity, where members share both explicit safety protocols and tacit experiences without immediate quid pro quo (72). This relational safety mitigates fears of knowledge misuse or undervaluation (73), while CG-enhanced psychological safety (74) and informal networks (75) further lower barriers to knowledge flow. Besides, from the lens of Nonaka’s SECI model (76), tacit knowledge transfer relies on “socialization”—direct, informal interactions. CG acts as a social infrastructure for this process, particularly in high-risk environments where tacit knowledge (e.g., safety practices) is embedded in personal routines (77, 78). By fostering dense interpersonal networks and frequent face-to-face exchanges, CG enables the diffusion of context-dependent, hard-to-codify knowledge (79). This aligns with Nonaka’s emphasis on relational trust as a prerequisite for tacit knowledge sharing, where shared understanding and contextual familiarity reduce the need for formal codification. Thus, CG operationalizes the SECI model’s “socialization” phase by creating the relational conditions necessary for tacit knowledge to emerge and circulate within teams.

High levels of TKS significantly enhance WSBs by operationalizing core principles of knowledge sharing theory. According to Nonaka’s SECI model (76), tacit knowledge transfer relies on informal interactions. In safety-critical environments like construction, TKS facilitates the diffusion of context-dependent, hard-to-codify safety practices (e.g., hazard recognition or emergency response) through dense interpersonal networks (80). In addition, TKS reduces knowledge stickiness—the difficulty in transferring complex or implicit knowledge (81). By fostering shared mental models and contextual understanding, TKS enables teams to interpret and apply safety knowledge more effectively (82). Chen et al. (83) highlighted that TKS interventions in construction settings improve proactive risk identification by aligning team members’ cognitive frameworks. Besides, TKS strengthens collective responsibility for safety by aligning cognitive frameworks (79). When teams share both technical standards and personal safety experiences, members develop a shared sense of accountability, perceiving safety as a collective obligation rather than an individual task. This aligns with Edmondson’s (84) concept of psychological safety, where open communication and trust encourage risk-disclosure and behavior adoption. Yang et al. (85) further demonstrated that TKS enhances safety initiative participation by fostering empowerment and collaborative problem-solving, as evidenced by increased engagement in hazard reporting and preventive measures. According to the previous argument, follow-up hypotheses can be proposed.

*H5*: Coworker guanxi is associated with team knowledge sharing.

*H6*: Team knowledge sharing is associated with workers’ safety behavior.

*H7*: Team knowledge sharing mediates the relationship between coworker guanxi and workers’ safety behavior.

### 2.4 The moderating effect of team safety climate

TSC refers to employees’ shared perceptions of the priority of safety within their team (27, 28). The focus on the TSC dates back to Zohar’s observations that when compared to organizational safety climate, the TSC predicts more safety outcomes at the individual level in the manufacturing industry (27). Lingard et al. (28, 29) introduced the discussion of this concept into the construction industry because of its salient role in shaping workers’ safety behaviors in this loosely structured industrial sector. The TSC can be reflected in the extent to which team supervisors and coworkers prioritize safety, so two conceptualized approaches exist in the existing studies. The concept can be a unicomponent variable (supervisor’s safety response) (27) and a dual-component variable (i.e., supervisor safety response and coworkers’ safety responses) (86).

TSC denotes the degree to which safety is prioritized by supervisors and coworkers. In high-level TSC, the supervisor may facilitate safety-related communication by organizing safety-related meetings (87), and the coworkers may voluntarily share their safety-related experiences and help others to handle safety problems (88). Thus, these interactions will strengthen the level of CG and its predictive ability for other variables (e.g., TI and TKS). Besides, we argue that TSC can significantly affect TI. The frontline workers are more vulnerable to safety accidents. In stronger TSC, supervisors are often observed concerning the onsite safety management, such as providing enough and high-quality PPEs, and regularly carrying out safety inspections (28), the frontline workers accordingly note that their supervisors care about their safety, thus they will enhance their identification with the team (89, 90). In addition, TSC can also influence the TKS. On one hand, as previously argued, the team supervisor will develop measures to facilitate the safety-related communication (e.g., the periodic safety meetings and mentorship) when the team has a higher TSC (91). On the other hand, TSC can also reflect workers’ trust in the team’s priority on safety (92), this trust is the prerequisite for explicit and tacit knowledge sharing among workers. Based on the aforementioned analyses, we can presume:

*H8*: Team safety climate can moderate the relationship between coworker guanxi and team identification;

*H9*: Team safety climate can moderate the relationship between coworker guanxi and team knowledge sharing.

**Figure 1:**
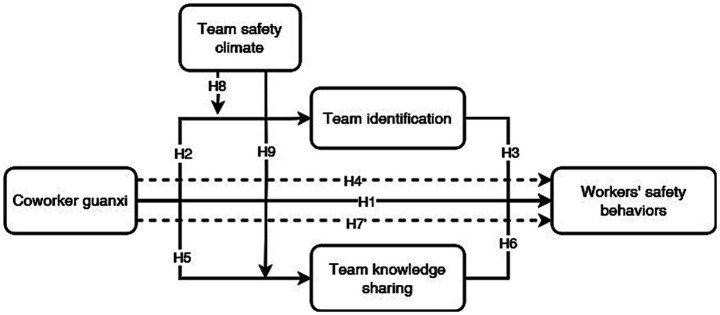
The conceptual model of this research.

Based on the above hypotheses, a conceptual model is developed as shown in Figure 1.

## 3 Research methodology

### 3.1 Survey procedure and participants

Convenience sampling is widely used in construction worker-related research (93, 94), because the construction workers are not easy to contact (95). Therefore, we chose this sampling approach to select the survey participants. Full-time construction workers, excluding managers, in Zhengzhou, Changsha and Xi’an were selected to gather empirical data due to pre-established collaborations with construction enterprises in these cities, which grant us survey access to construction workers. This survey method can help to improve the reliability of the survey data because of these managers’ support (94). Besides, to enhance representativeness, we ensured coverage across key project types: residential, infrastructure, and commercial. The survey was conducted using online questionnaire, which includes there sections: section A presents an electronic informed consent form, section B provides questions regarding the participants’ demographic details (e.g., gender, age, educational level, tenure, and working time in current team); section C includes the measurement questions for CG, TI, TKS, WSB, and TSC. We used the wenjuanxing as an online questionnaire platform (93, 96). The online questionnaire was initially transferred to selected construction managers, and then shared with the full-time construction workers they supervise. To ensure respondent anonymity, surveys do not require personally identifiable information. Participants were informed by the electronic informed consent form that their responses were confidential and would be used solely for academic research purposes. The subsequent questionnaire can only be conducted after the workers agree to the electronic informed consent form. The survey period is from October 2023 to December 2023, and a total of 389 questionnaires were collected, with 347 deemed valid for follow-up analyses. Demographic information of the participating workers was presented in Table 1.

**Table 1:** Demographic details of the participant construction workers.

Variables	Options	Numbers	Percentage (%)
Gender	Male	323	93.1
	Female	24	6.9
Age	20 or younger	13	3.75
	21 to 30	73	21.04
	31 to 40	75	21.61
	41 to 50	137	39.48
	50 or elder	49	14.12
Educational level	Primary school or below	45	12.97
	Junior high school	167	48.13
	Senior high school or same level	96	27.67
	College or above	39	11.24
Tenure	Less than 1	30	8.65
	2 to 5	118	34.01
	6 to 10	127	36.60
	More than 10	72	20.75
Working time in current team	Less than 1	23	6.63
	2 to 5	162	46.69
	6 to 10	105	30.26
	More than 10	57	16.43
Project type	Residential project	158	45.53
	Infrastructure	132	38.04
	Commercial project	57	16.43

### 3.2 Measurement instruments

The measurement scale of CG was developed according to Chen et al.’s research (35). The scale includes 8 items that assess the two components of CG, i.e., affective attachment (AT) and personal-life inclusion (PLI). An example item is “My workmates and I always share thoughts, opinions, and feelings toward work and life”.

TI was evaluated using the measure scale developed by Van Der Vegt and Stuart Bunderson (47). The scale consists of 4 items, an instance of the item is “I feel emotionally attached to my work team”.

TKS was assessed using the measure scale validated by Sang et al. (68). There exist 9 items in this scale, with 4 items for explicit knowledge sharing (EKS) and 5 items for tacit knowledge sharing (AKS). An example question is “the older members of the team will share safety expertise and special skills”.

The measurement scale of TSC was from Zhang et al.’s research (23). The scale has 6 items, and one example item is that “safe working is a condition of employment in our workteam”.

WSB was assessed using the scale introduced by Neal and Griffin (39), comprising 6 items across two components: safety compliance and safety participation. A typical example is: “I comply with all safety rules at work”.

All variables were rated on a five-point Likert scale, from 1 (strongly disagree) to 5 (strongly agree).

Several variables were selected as control variables because of their latent effects on WSB. These variables include workers’ gender (0 for male, 1 for female), educational level (0 for primary school or below; 1 for junior high school, 2 for senior high school or same level, 3 for college or above), and tenure (0 for less than 1, 1 for 2 to 5, 2 for 6–10, 3 for more than 10).

### 3.3 Data analysis methods

Several statistical techniques were employed to handle the survey data. The collected data were first imported into SPSS to test normality and reliability; the indices include skewness, kurtosis, and Cronbach’s alpha.

Subsequently, the remaining data were imported into AMOS 23 for confirmatory factor analysis (CFA). Six measurement models, including the hypothesized model and 5 other alternative models, were established for CFA to test the structural validity of the measurement. Convergent and discriminant validity were evaluated using factor loadings (FL), composite reliability (CR), and average variance extracted (AVE).

Then, the covariance-based latent variable model (LVM) was selected to test the hypothesized relationships using AMOS 23.0. LVM was chosen for three key reasons: (1) it allows simultaneous estimation of multiple relationships involving latent constructs, which fits our complex mediation-moderation model; (2) it accounts for measurement errors in observed variables, enhancing parameter accuracy; (3) it supports the evaluation of both direct and indirect effects through bootstrapping, which is essential for testing mediation hypotheses (97).

Model fit was assessed using multiple indices: *χ*^2^/df (acceptable if <5, good if <3), goodness fit index (GFI > 0.8 acceptable), comparative fit index (CFI > 0.8 acceptable) and Root Mean Square Error of Approximation (RMSEA <0.08 acceptable) (98). These thresholds were used to determine whether the models adequately represented the observed data.

For mediation testing (H4, H7), we used bias-corrected bootstrapping with 5,000 resamples to calculate 95% confidence intervals for indirect effects. Significant mediation is established if the confidence interval excludes zero (99). For moderation analysis (H8, H9), interaction terms (CG × TSC) were created using item packing technique before including them in the LVM model framework (100).

## 4 Research results

### 4.1 Reliability and validity

The observed data were input into SPSS 23 for analysis of skewness and kurtosis. The results indicated that skewness values ranged from 0.04 to 1.58 (all below the threshold of 3), and the kurtosis values ranged from 0.03 to 2.23 (all below the threshold of 10). These statistical characteristics indicate that the observed data conform to a normal distribution.

Given the data were collected from three Chinese cities, the data were then subjected to post-hoc ANOVA analyses to evaluate the regional disparity. The tests show no regional disparity was found because the between-group variances of key variables (CG, TI, TKS, TSC and WSB) are not significant with *p*-values ranging from 0.162 to 0.484 (*p* > 0.05).

To assess the data’s suitability for factor analysis, the Kaiser-Meyer-Olkin (KMO) measure and Bartlett’s test of sphericity were performed. KMO values for all variables exceeded 0.70, and Bartlett’s test showed significant results (*p* < 0.05), confirming that the dataset met the assumptions required for further exploratory and confirmatory analyses.

Moreover, Harman’s one-factor test was performed to evaluate the common method variance. The results show that the first principal factor only explains 17.89% of the total variance (<40%), which suggests that common method bias is not significant.

CFA was carried out using AMOS 23 to assess the structural validity of the measurement scales. Six measurement models (including the hypothesized model and 5 alternative models) were established for CFA, and the calculated results were presented in Table 2. As presented, our hypothesized model demonstrates a better fit (*χ*^2^/df = 3.01, GFI = 0.857, CFI = 0.872, RMSEA = 0.061). According to the CFA results of the hypothesized model, the values of FL, CR and AVE were obtained, which were presented in Table 3. We removed 7 measurement items because the FL values of these items are less than 0.5 (98), of which 2 items for CG, 2 items for TKS, 1 item for TSC, and 2 items for WSB. Besides, the square roots of the AVE for each variable were compared to their correlations with other variables, and the results were shown in Table 4. The comparison results demonstrated that the measurement had better discriminant validity.

**Table 2:** Results of the CFA for the six models.

Models	*χ*^2^/df	GFI	CFI	TLI	RMSEA
Five-factor hypothesized model (including CG, TI, TKS, WSB and TSC)	3.01	0.857	0.872	0.883	0.061
Four-factor model 1 (TSC and TKS were merged into one factor)	4.71	0.781	0.802	0.813	0.079
Four-factor model 2 (TSC and TI were merged into one factor)	5.43	0.741	0.787	0.793	0.092
Three-factor model (TSC, TI, and TKS were merged into one factor)	7.61	0.703	0.721	0.723	0.104
Two-factor model (CG, TSC, TI and TKS were merged into one factor)	10.23	0.621	0.634	0.627	0.139
One-factor model	12.31	0.574	0.589	0.579	0.159

**Table 3:** Analysis of the CV of the measurement scale.

Variables/dimensions		Measurement item	Factor loading	Reliability	CR	AVE
CG	AT	AT1	0.779	0.81	0.747	0.498
		AT2	0.722	0.79		
		AT4	0.607	0.83		
	PLI	PLI1	0.832	0.79	0.725	0.474
		PLI3	0.633	0.83		
		PLI4	0.574	0.84		
TKS	EKS	EKS1	0.753	0.91	0.770	0.528
		EKS2	0.749	0.84		
		EKS3	0.675	0.82		
	AKS	TKSA1	0.813	0.89	0.797	0.500
		TKSA2	0.755	0.87		
		TKSA3	0.677	0.79		
		TKAS4	0.557	0.77		
TI		TI1	0.804	0.89	0.822	0.537
		TI2	0.755	0.91		
		TI3	0.737	0.83		
		TI5	0.642	0.87		
TSC		TSC1	0.743	0.84	0.832	0.499
		TSC2	0.751	0.81		
		TSC3	0.721	0.85		
		TSC4	0.677	0.86		
		TSC5	0.934	0.89		
WSB		WSB1	0.813	0.89	0.851	0.589
		WSB2	0.754	0.92		
		WSB3	0.786	0.88		
		WSB6	0.711	0.86		

**Table 4:** Discriminant validity analysis of the measurement scale.

Variables	AT	PLI	TI	EKS	AKS	TSC	WSB
AT	0.670*						
PLI	0.613	0.688*					
TI	0.489	0.534	0.733*				
EKS	0.378	0.346	0.321	0.727*			
AKS	0.412	0.409	0.334	0.609	0.707*		
TSC	0.229	0.311	0.246	0.223	0.241	0.706*	
WSB	0.489	0.437	0.409	0.454	0.489	0.439	0.767*

### 4.2 Test of main effects

LVM1 was established to evaluate the main effect of CG and WSB, of which CG was set as the explanatory variable. The results calculated based on the remaining data demonstrate a satisfactory fit, with *χ*^2^/df = 2.89, GFI = 0.826, CFI = 0.847, and RMSEA = 0.049. Table 5 illustrates the path coefficient and its statistical significance. As can be seen, the path coefficient is significant, indicating there is a salient association between CG and WSB, with a main effect size = 0.601. As such, Hypothesis H1 is supported.

**Table 5:** Path coefficient and significance of LVM1.

Model	Path	Path coefficient	S. E.	C. R.	*p*
LVM1	CG → WSB	0.601	0.108	8.74	***

“***”Indicates that the value is less than 0.001.

### 4.3 Test of the dual mediating effects

Latent variable model 2 (LVM 2) was established to assess the dual mediating effects of TI and TKS in the relationship between CG and WSB. After the calculation based on the collected data, this model shows a satisfactory fit with the test indices significant. (*χ*^2^/df = 3.14, RMSEA = 0.065, GFI = 0.814 CFI = 0.819). The path coefficients and their significance levels are presented in Table 6, while the estimates and significance of the mediating effects are summarized in Table 7.

**Table 6:** Significance analysis of LVM2 path coefficients.

Models	Path	*β*	S. E.	C. R.	Bootstrap 5,000				*p*
					Bias-corrected		Percentile		
					Lower	Upper	Lower	Upper	
LVM 2	TI ← CG	0.494	0.109	6.177	0.344	0.603	0.356	0.620	***
	WSB ← TI	0.438	0.085	6.238	0.258	0.537	0.254	0.530	***
	TKS ← CG	0.523	0.112	6.213	0.344	0.632	0.385	0.649	***
	WSB ← TKS	0.439	0.084	6.187	0.260	0.539	0.256	0.532	***
	WSB ← CG	0.155	0.105	2.87	0.077	0.352	0.105	0.338	***

“***”Indicates that the value is less than 0.001.

**Table 7:** Significance of LVM2 mediating effect.

Effect type	Path coefficients	S. E.	C. R.	Bootstrap 5,000				*p*
				Bias-corrected		Percentile		
				Lower	Upper	Lower	Upper	
WSB ← TI ← CG	0.216	0.117	3.71	0.229	0.437	0.231	0.412	***
WSB ← TKS ← CG	0.230	0.121	3.79	0.254	0.463	0.256	0.441	***
Total indirect effect	0.446	0.119	3.75	0.242	0.650	0.244	0.560	***
Direct effect	0.155	0.105	2.87	0.077	0.352	0.105	0.338	***
Total effect	0.601	0.108	8.74	0.575	0.962	0.625	0.925	***

“***”Indicates that the value is less than 0.001.

After 5,000 bootstrap samples, the path coefficients for TI ← CG and WSB ← TI were 0.494 and 0.438, respectively, both statistically significant (*p* < 0.001). These results suggest that positive CG actively promotes TI, and enhanced TI further positively influences WSB. Thus, Hypotheses H2 and H3 are supported. Consequently, it can be inferred that TI mediates the relationship between CG and WSB, confirming Hypothesis H4. As shown in Table 7, CG indirectly and positively affects WSB through TI, with an effect size of 0.216.

Additionally, after 5,000 bootstrap samples, the path coefficients for TKS ← CG and WSB ← TKS were 0.532 and 0.439, respectively, both of which were statistically significant (*p* < 0.001). The findings suggest that favorable CG facilitates TKS, and TKS further positively influences WSB. Therefore, Hypotheses H5 and H6 are validated. It follows that TKS mediates the relationship between CG and WSB, supporting Hypothesis H7. As indicated in Table 7, CG indirectly and positively influences WSB through TKS, with an effect size of 0.230.

According to Tables 6, 7, the direct path coefficient from CG to WSB in LVM2 is 0.155 and statistically significant, indicating that CG has a direct and positive impact on WSB, with a direct effect size of 0.155. Both TI and TKS serve as mediators in this relationship. Comparing the indirect effects of the two mediation paths, it is evident that the mediating effect of TKS is slightly stronger than that of TI.

### 4.4 Test of the moderation effect

To assess the moderation effects of TSC in the relationship between CG and TI, and the relationship between CG and TKS, two other LVMs (LVM 3 and LVM 4) with the interaction term (i.e., CG × TSC) were established. Based on the calculation with the gathered data, the fit indices are presented in Table 8, and the path coefficients and their significance were presented in Table 9.

**Table 8:** Results of LVM 3 and LVM 4.

Models	Specification of the model	*χ*^2^/df	GFI	CFI	TLI	RMSEA
LVM3	CG, TSC, and CG × TSC are the explanatory variables, TI is the explained variable.	2.78	0.811	0.827	0.819	0.063
LVM4	CG, TSC, and CG × TSC are the explanatory variables, TKS is the explained variable.	2.53	0.831	0.847	0.834	0.059

**Table 9:** Path coefficients significance analysis of LVM3 and LVM4.

Models	Path	*β*	S. E.	C. R.	Bootstrap 5,000				*p*
					Bias-corrected		Percentile		
					Lower	Upper	Lower	Upper	
LVM 3	TI ← CG	0.287	0.095	3.02	0.213	0.457	0.247	0.47	***
	TI ← TSC	0.207	0.102	2.30	0.157	0.432	0.167	0.421	***
	TI ← CG × TSC	0.102	0.108	1.02	0.052	0.152	0.062	0.142	***
LVM 4	TKS ← CG	0.313	0.089	3.13	0.263	0.489	0.273	0.453	***
	TKS ← TSC	0.216	0.122	2.27	0.166	0.421	0.176	0.403	***
	TKS ← CG × TSC	0.114	0.126	1.09	0.064	0.164	0.074	0.154	***

“***”Indicates that the value is less than 0.001.

**Figure 2:**
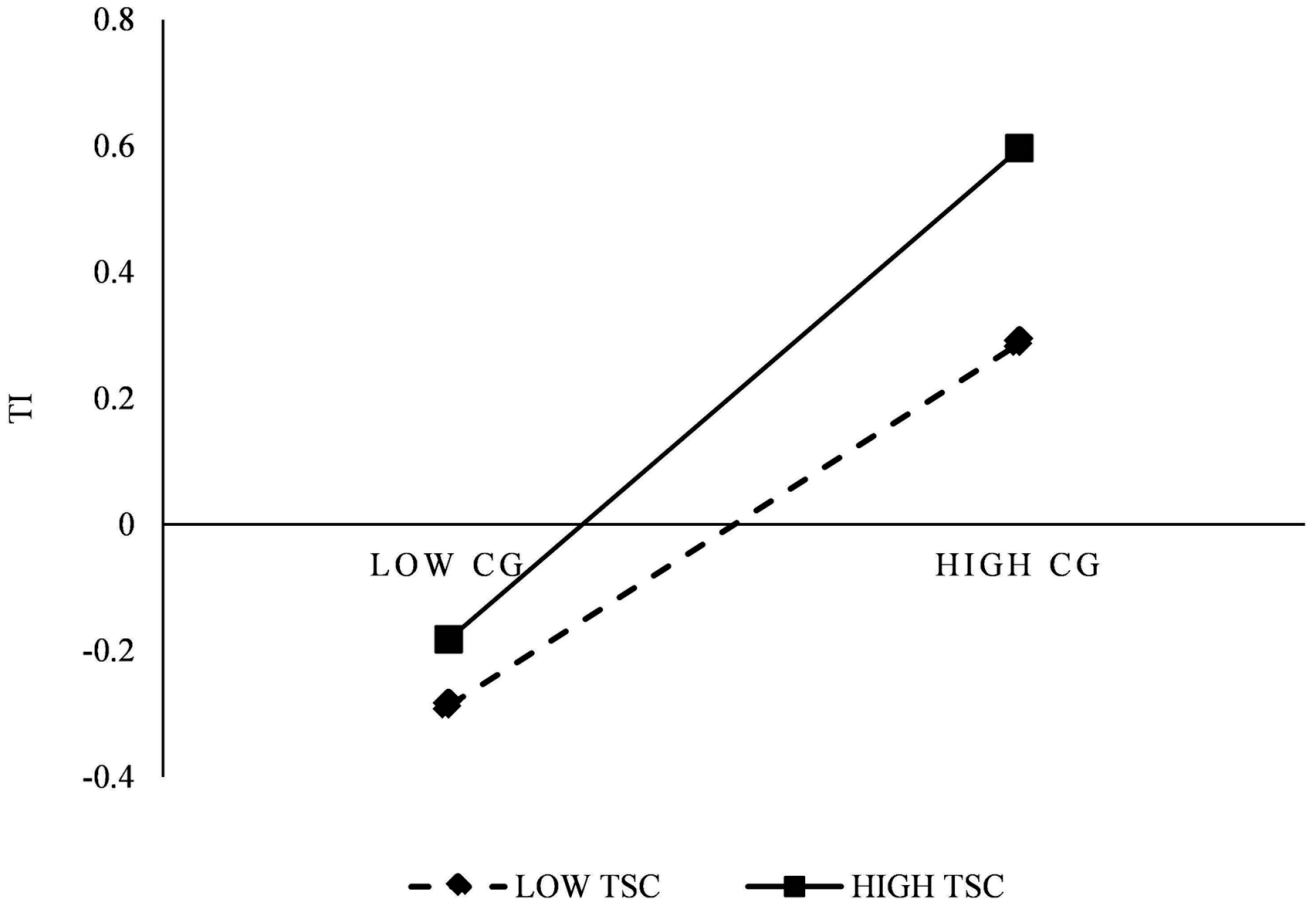
The moderation effect of TSC on CG and TI.

**Figure 3:**
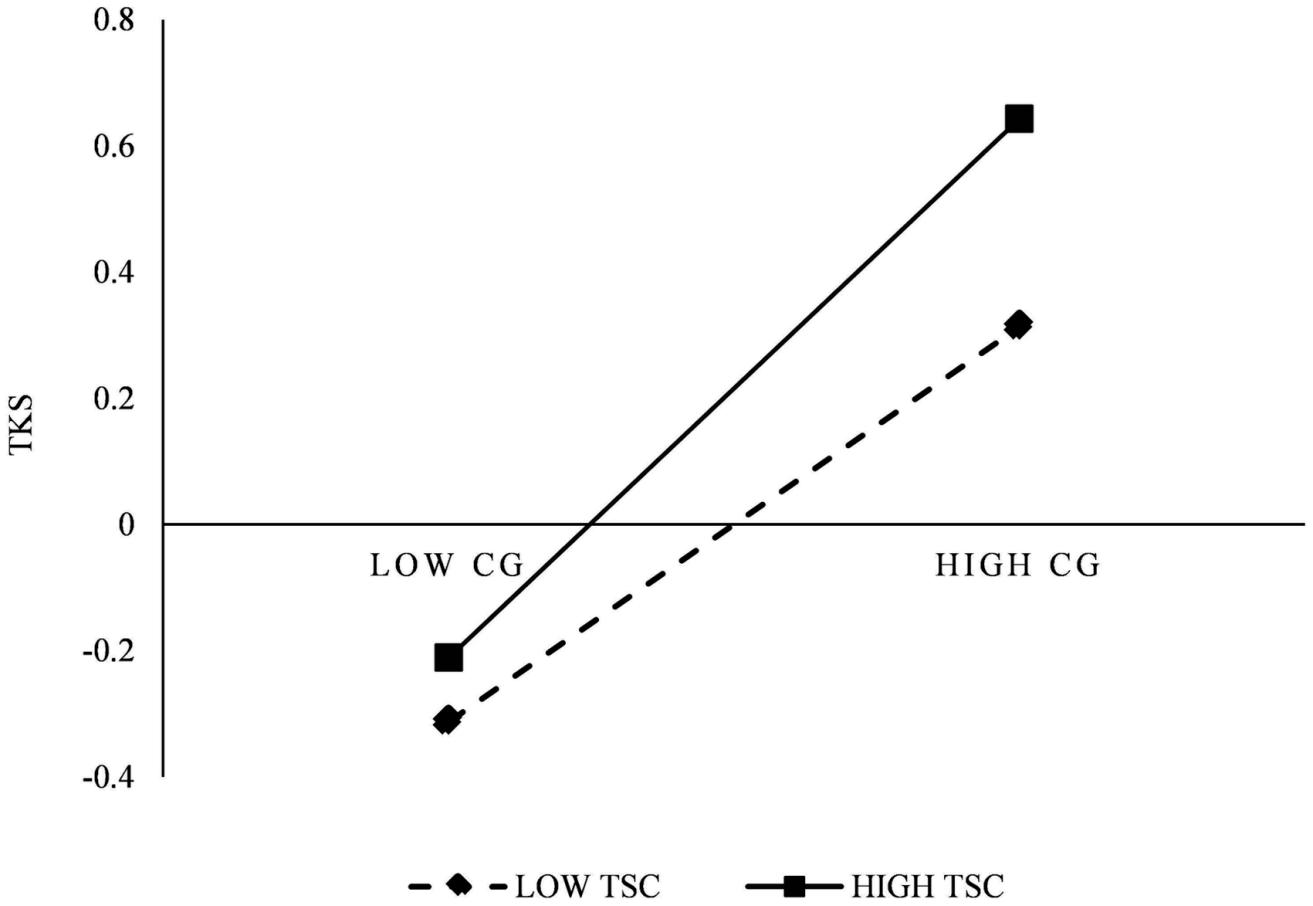
The moderation effect of TSC on CG and TKS.

As can be seen in Tables 8, 9, LMV3 has a satisfactory fit (*χ*^2^/df = 2.78, GFI = 0.811, CFI = 0.827 and RMSEA = 0.063), and the path coefficient is equal to 0.102 and significant (*p* < 0.001). Figure 2 presents the moderation effect of TSC on the relationship between CG and TI. These results demonstrated that the TSC has a significant moderation effect on the relationship between CG and TI. As such, hypothesis H8 was supported. Besides, LMV4 also show a satisfactory fit (*χ*^2^/df = 2.53, GFI = 0.831, CFI = 0.847 and RMSEA = 0.059), and the path coefficient is equal to 0.114 and significant (*p* < 0.001). Figure 3 shows the moderation effect of TSC on the relationship between CG and TKS. These results demonstrate the TSC has a salient moderation effect on the relationship between CG and TKS, as such, hypothesis H9 was supported.

## 5 Discussion and managerial implications

### 5.1 Discussion

#### 5.1.1 Influence of coworker guanxi on workers’ safety behavior

The research confirmed a significant relationship between coworker guanxi (CG) and workers’ safety behaviors (WSBs), with a robust path coefficient of 0.601 (*p* < 0.001). This aligns with Chen et al.’s (22) seminal work demonstrating behavioral impact of CG in Chinese construction settings. Besides, Zhang et al. (23) and Chen et al. (33) pointed out the safety-related interactions based on coworker relations can facilitate the safety knowledge sharing and foster a reciprocal safety obligations, further enhancing the adoption of safety behaviors among construction workers. Additionally, our research is also a practical test of Bian’s guanxi theory in workers’ safety behaviors, which effectively explains that guanxi-based trust and emotional closeness in work teams generate moral responsibilities (8).

However, compared to Chen et al.’s research (22), which treated CG as a unidimensional construct, our research fundamentally advances beyond the existing literature by dissecting CG into affective attachment (AT) and personal-life inclusion (PLI). Our research highlighted that, except for AT, which is characterized by emotional interdependence and is often investigated in prior studies (10, 11), PLI, which focuses on life-domain intercourse, can also exert a salient effect on an individual’s behavior performance. Besides, we further imply that AT (average factor loading = 0.703) drives a stronger direct safety effect than PLI (average factor loading = 0.680). This difference in effect size between the two dimensions can also be explained. Compared to AT, the PLI is a more distal antecedent because high level of PLI with workmates often lead to high-level AT in the current Chinese society.

Western frameworks often concentrate on formal safety programs or individual incentives, but our research argues that multi-dimensional dynamics inherent in coworker guanxi can also significantly influence the WSB. The reason is that workers feel morally obligated to reciprocate the trust and support from their workmates. As such, this study extends the application of social exchange theory (43) in Chinese workers’ safety behavior management. Further, Zohar (101) insight on informal networks supports our finding: dominance of AT in safety behaviors reflects how emotional bonds (vs. generalized PLI ties) create faster-acting social accountability, a phenomenon amplified in China’s guanxi-permeated construction sector.

#### 5.1.2 Mediation effect of team identification

The research confirmed team identification (TI) significantly mediated the CG-WSB relationship (indirect effect = 0.216, *p* < 0.001). This result reinforces Chen et al.’s (22) conclusion that shared team identity transforms relational ties into workers’ safety practices. This aligns with social identity theory (47): coworker guanxi-based interaction among construction workers can increase the identification with the team (102), and when the workers emotionally identify with their team, they internalize collective safety norms as personal values, reducing at-risk behaviors. Moreover, Chen et al. (33) argued that the guanxi with supervisor and workmates often denotes the same region and culture, which is the base of mutual identification; and this mutual identification is more likely to internalize team values, including safety-related norms, and engage in safety citizenship behaviors (22, 33, 60).

This study further extend the understanding of TI in relation to safety behavior in construction by integrating the two-dimensional CG into the model. When compared to Chen et al.’s (22) study, our research also indirectly illustrates that TI can mediate the relationships between AT, PLI and WSBs. This result fundamentally advances beyond the previous studies by providing a more in-depth explanation of how TI functions in the relationship between CG and WSBs. Besides, our research can also imply that AT exert a stronger effect on TI than PLI. As argued earlier, the research provides more in-depth practical references for promoting construction workers’ TI.

This study deepens the understanding of organization identification theory (103) by showing how CG and its dimensions (AT and PLI) can serve as a facilitator of TI in a collectivist context like China’s construction industry. Traditional views on organizational identification have focused on cognitive and emotional processes that align employees with organizational values. However, this study shows that coworker guanxi, grounded in reciprocal relationships, also influences workers’ identification with their teams, highlighting the role of relational dynamics in organizational behavior.

#### 5.1.3 Mediation effect of team knowledge sharing

The results further suggest that TKS mediates the relationship between CG and WSB, aligning with existing literature that highlights the role of knowledge sharing in improving organization expected behaviors. Previous studies indicate that effective TKS can increase workers’ awareness of hazards and best practices, and further enhances workers’ safety outcomes. For example, Zhang et al. (23) pointed out that workers in teams with strong TKS are more likely to adopt safety behaviors because they better understand safety protocols. Besides, Mei et al. (79) and Yang et al. (85) also argued that sharing safety-related knowledge leads workers to follow regulations and helps to establish a safer work environment.

This study incorporates CG into the framework of TKS and WSB, thus expanding the existing literature. We demonstrate that guanxi is a culturally embedded mechanism and can play a key role in boosting TKS. In a collectivist society like China, guanxi creates an environment where trust and mutual obligations motivate workers to share critical safety knowledge with their peers. As Huang et al. (104), Ding et al. (74) and Ren et al. (105) argued, guanxi can increase trust and foster open communication and knowledge exchange, which is essential for improving safety behaviors on construction sites. This study, therefore, adds a cultural dimension to our understanding of how interpersonal relationships influence safety behavior through knowledge sharing.

From a theoretical perspective, this study contributes to knowledge sharing theory (76) by extending it to the context of safety behavior in construction settings from the aspect of guanxi. This study shows that CG enhances both the quality and frequency of safety knowledge sharing. By integrating relational dynamics into the theory, this research emphasizes the importance of social ties in facilitating the exchange of knowledge, particularly in environments where timely and accurate information can directly prevent accidents. This study, therefore, highlights how informal social networks like guanxi contribute to the transfer of safety-related knowledge and the improvement of safety behaviors.

#### 5.1.4 Moderation effect of team safety climate

The results show that TSC moderates the link between CG and both TI and TKS. These findings are aligned with the existing literature. The previous studies argued the complicated roles of TSC in facilitating WSB in the construction industry. For instance, Zhao and Li (106) found that a positive safety climate fosters a cohesive team environment, which enhances workers’ psychological resilience and promotes safety behavior. Similarly, Lingard et al. (29) observed that a strong safety climate encourages cooperation and mutual support among workers, which is vital for TI and effective TKS.

Previous research on TSC mainly focused on its direct impact on safety behavior or examined the single-path moderation mechanism (39, 101). This study contributes to the literature by exploring the dual-path moderating effect of TSC on the relationships between CG, TI, and TKS, and thus offers a more comprehensive approach in conceptualizing the effect of TSC in the construction industry. Additionally, this study emphasizes the moderating role of TSC on informal or indigenous guanxi-related interaction process in behavioral motivation in the Chinese construction industry, i.e., by influencing TI and TKS. The TSC’s moderating effect on such informal relational dynamics offers a novel contribution, suggesting that safety behaviors in high-risk environments are influenced not only by formal structures but also by the social and cultural dynamics that underpin team interactions.

The theoretical contribution of this study lies in its further extension of social exchange theory (43) to the context of TSC and CG. By highlighting the moderating role of TSC, this research underscores how relational dynamics within teams can be strengthened in environments that emphasize safety. In high-level TSC, workers are more inclined to cooperate, share safety knowledge, and develop a strong team identification.

#### 5.1.5 Cultural context and generalizability of findings

While the present study was conducted in China, where guanxi plays a critical cultural role, it is important to consider whether and how these findings might transfer to other cultural contexts. Guanxi, rooted in Confucian traditions, emphasizes reciprocal obligations, affective trust, and long-term interpersonal bonds, which are particularly salient in collectivist societies (8, 9). In individualistic cultures, such as those prevalent in North America or Western Europe, CG may be less embedded in personal-life inclusion and more influenced by formal contracts or professional boundaries. Consequently, the pathways identified in this study, particularly the strong mediating roles of TI and TKS, might manifest differently, as interpersonal trust may rely more on professional competence than on affective attachment.

Moreover, in societies with low-context communication styles, safety knowledge sharing may occur more through formal channels and explicit procedures, potentially weakening the informal relational mechanisms emphasized in our model. However, elements of our framework could still apply in other collectivist or relationship-oriented cultures, such as in Asia or Latin America, where trust-based CG are also prominent (9, 107, 108). Future cross-cultural comparative studies could empirically examine how cultural dimensions (e.g., individualism–collectivism, power distance, uncertainty avoidance) moderate the CG–WSB relationship, thereby refining the applicability of this model beyond China. This would provide a richer understanding of whether guanxi-like relational mechanisms are universally relevant for improving safety behavior, or whether they are culturally bounded.

### 5.2 Management implications

This study highlights the critical role of coworker guanxi (CG) in enhancing safety behaviors within the construction industry. By focusing on the two dimensions of coworker guanxi (affective attachment and personal-life inclusion), managers can create a more supportive and safety-oriented work environment. The following actionable strategies are proposed to help Chinese construction managers foster positive coworker relationships and improve workers’ safety behaviors.

Firstly, fostering affective attachment through team-building activities. Affective attachment, which involves strong emotional bonds between workers, is essential for cultivating trust and mutual responsibility. Managers should prioritize team-building activities that focus on emotional connections and mutual support. These activities can include collaborative safety exercises, team-based problem-solving workshops, and social events. By enhancing emotional interdependence among workers, Affective attachment can increase accountability and safety behavior, as workers are more likely to look out for each other’s well-being (10).

Secondly, encouraging personal-life inclusion (PLI) through informal interactions or intercourse. Personal-life inclusion refers to the sharing of personal experiences and life outside of work, which strengthens interpersonal ties. Managers can create informal spaces for workers to share their personal stories, celebrate milestones, and interact in a relaxed environment. This might involve social gatherings, regular lunch breaks, or informal safety meetings. When workers feel included in each other’s personal lives, it fosters a sense of belonging and increases their commitment to mutual safety goals. This practice helps create a cohesive team where workers support one another not only professionally but also personally.

Thirdly, promoting team identification (TI) to align safety goals. Enhancing team identification is crucial for ensuring that workers align their behaviors with team safety goals. Managers should encourage team identification by involving workers in safety goal-setting processes and regular safety workshops. Encouraging workers to share their experiences and solutions during these sessions helps reinforce collective responsibility for safety. When workers feel emotionally connected to their team, they are more likely to internalize the team’s safety values and follow safety protocols (22).

Fourthly, fostering team knowledge sharing (TKS) for safety awareness. Facilitating the exchange of safety-related knowledge among coworkers enhances overall safety compliance. Managers should encourage knowledge-sharing practices by creating platforms for workers to discuss safety challenges and solutions. This could include safety meetings where workers are encouraged to share personal experiences or safety tips. Strong coworker relationships, particularly those based on AT and PLI, create an environment where workers feel comfortable sharing both tacit and explicit safety knowledge, thereby improving team safety outcomes (16).

Fifthly, developing a positive team safety climate. A positive safety climate amplifies the effects of coworker guanxi on safety behaviors. Managers should focus on creating a team safety climate where safety is prioritized by both supervisors and coworkers. Regular safety meetings, team-building exercises, and clear communication from management about safety expectations can reinforce the importance of safety and strengthen the bond between workers. A positive TSC enhances the effects of AT and PLI, as it provides a supportive framework for workers to share knowledge and support each other in adhering to safety protocols.

Finally, while these strategies are rooted in the Chinese cultural context, they can be adapted to international environments with attention to cultural differences. In multicultural teams, managers should be mindful of how coworker relationships, team dynamics, and communication styles vary across cultures. For instance, affective attachment and personal-life inclusion may have different significance in individualistic versus collectivist cultures. To ensure effectiveness, managers should incorporate cross-cultural training, encourage intercultural communication, and adapt strategies to fit the values and norms of diverse teams. By considering these cultural nuances, managers can foster strong coworker relationships and improve safety behaviors across global teams.

### 5.3 Limitations and future research

Although this study offers insightful information about how CG affects WSBs, it must be noted that the study has several limitations. First, the research’s cross-sectional design makes it more difficult to determine causality. A more thorough grasp of how CG, team dynamics, and safety behavior change over time would be possible with a longitudinal approach. To investigate these relationships further and look at the long-term impacts of coworker relationships and team safety climate on safety behaviors, future research could use experimental or longitudinal designs.

Second, social desirability bias could be introduced by depending too much on self-reported data, especially in collectivist cultures where people might feel pressured to report positive behaviors. Future studies should use data from multiple sources, including peer assessments, supervisor evaluations, and direct safety observations, to improve the validity of the findings and provide a more complete picture of employees’ safety practices. A stronger link between subjective safety perceptions and real safety measures on the job site may also be made possible by incorporating behavioral safety data.

Third, future research could investigate other contextual factors that affect the relationship between CG and WSB. Individual elements like psychological safety, personal attitudes toward safety, and job stress, for instance, can have a big impact on WSB. It would also be beneficial to look into how leadership styles influence team safety climate and safety behavior. Analyzing the effects of various leadership philosophies, such as participative or paternalistic leadership, on relationships among coworkers and the safety environment may provide useful information for enhancing safety management.

Fourth, while our study focused on TSC’s moderating role between CG and TI/TKS pathways, it can be argued that TSC may also moderate other relationships (e.g., between TI and WSB or TKS and WSB), or even be strengthened by TI/TKS as an outcome. Future research can explore these alternative pathways through longitudinal designs or agent-based simulations to map dynamic interactions among CG, TI, TKS, and TSC.

Finally, this study was carried out in China, where guanxi is especially significant. Evaluating the findings’ generalizability would involve comparing them in various cultural contexts. Future studies could look into how CG affects WSB in nations with various organizational structures and cultural norms, especially in sectors like construction, where safety is essential.

## 6 Conclusion

This study investigated how CG affected WSBs in the Chinese construction sector, with particular attention to the moderating influence of TSC and the mediating functions of TI and TKS. Through the development and testing of a conceptual model, the study offers new perspectives on the social dynamics that underlie safety practices in construction teams, especially when considering guanxi, a significant aspect of Chinese culture.

The results demonstrate that CG has a significant impact on WSBs and that CG has a positive effect on both TI and TKS, which in turn improve safety behaviors. The study also emphasizes the moderating role of TSC, whereby a favorable safety climate enhances the connections among CG, TI, and TKS, thereby increasing WSB on construction sites.

In theory, this study makes a contribution to the existing literature by incorporating CG into the framework of WSB, thereby extending social exchange theory and deepening our understanding of how the dynamics of relationships within teams influence safety outcomes.

Practically speaking, the study emphasizes how crucial it is to develop a strong CG and a positive TSC in order to enhance safety behaviors. To increase employees’ dedication to safety and lower accident rates, managers should place a high priority on team-building, open communication, and culturally appropriate safety measures. In conclusion, this research emphasizes the role of relational dynamics and team-level factors in shaping safety behaviors in construction, offering both theoretical insights and practical recommendations for improving safety management.

## Data Availability

The original contributions presented in the study are included in the article/supplementary material, further inquiries can be directed to the corresponding author/s.
